# Suppressive Effects of Anthrax Lethal Toxin on Megakaryopoiesis

**DOI:** 10.1371/journal.pone.0059512

**Published:** 2013-03-21

**Authors:** Po-Kong Chen, Hsin-Hou Chang, Guan-Ling Lin, Tsung-Pao Wang, Yi-Ling Lai, Ting-Kai Lin, Ming-Chun Hsieh, Jyh-Hwa Kau, Hsin-Hsien Huang, Hui-Ling Hsu, Chi-Yuan Liao, Der-Shan Sun

**Affiliations:** 1 Department of Molecular Biology and Human Genetics, Tzu-Chi University, Hualien, Taiwan; 2 Institute of Medical Science, Tzu-Chi University, Hualien, Taiwan; 3 Department of Microbiology and Immunology, National Defense Medical Center, Taipei, Taiwan; 4 Institute of Preventive Medicine, National Defense Medical Center, Taipei, Taiwan; 5 Department of Obstetrics and Gynecology, Mennonite Christian Hospital, Hualien, Taiwan; University of Leuven, Belgium

## Abstract

Anthrax lethal toxin (LT) is a major virulence factor of *Bacillus anthracis*. LT challenge suppresses platelet counts and platelet function in mice, however, the mechanism responsible for thrombocytopenia remains unclear. LT inhibits cellular mitogen-activated protein kinases (MAPKs), which are vital pathways responsible for cell survival, differentiation, and maturation. One of the MAPKs, the MEK1/2-extracellular signal-regulated kinase pathway, is particularly important in megakaryopoiesis. This study evaluates the hypothesis that LT may suppress the progenitor cells of platelets, thereby inducing thrombocytopenic responses. Using cord blood-derived CD34^+^ cells and mouse bone marrow mononuclear cells to perform in vitro differentiation, this work shows that LT suppresses megakaryopoiesis by reducing the survival of megakaryocytes. Thrombopoietin treatments can reduce thrombocytopenia, megakaryocytic suppression, and the quick onset of lethality in LT-challenged mice. These results suggest that megakaryocytic suppression is one of the mechanisms by which LT induces thrombocytopenia. These findings may provide new insights for developing feasible approaches against anthrax.

## Introduction

*Bacillus anthracis*, the etiological agent of anthrax, is a Gram-positive, nonmotile, aerobic, spore-forming, rod-shaped bacterium [1]. Anthrax lethal toxin (LT) is a major virulence factor of *B. anthracis*, and it consists of two polypeptides: protective antigen (PA, 83 kDa) and lethal factor (LF, 90 kDa) [2]. LF is a zinc-dependent metalloprotease that cleaves the N-terminal domain of all the mitogen-activated protein kinase (MAPK) kinases (MKKs/MEKs) from MEK1 to MEK7, except MEK5 [3]. This disrupts three MAPK pathways downstream: the ERK (extracellular signal-regulated kinase), p38, and JNK (c-Jun N-terminal kinase) [4], [5]. LF is toxic only when combined with PA, a cellular receptor-binding component that delivers LF into cells [2], forming a lethal toxin (LT). PA binds to two known cell-surface receptors: tumor endothelium marker-8 (TEM8) and capillary morphogenesis protein-2 (CMG2) [6], [7].

LT challenge is sufficient to elicit mortality in various animal models [8], [9]. Although LT challenge may not reflect the full complexity of a *B. anthracis* infection, reductionist approaches using LT-treated cells and animals have enabled researchers to identify the pathogenic mechanism of LT. In cell culture and animal models, LT disrupts the functions of various cell types, including macrophages, lymphocytes, neutrophils, dendritic cells, endothelial cells, and platelets [10], and causes mortality in experimental mice and rats [8], [9], [11]. However, the pathogenic mechanism of LT in severe illness and death requires further clarification. Evidence indicates that once anthrax has induced septic shock, it will inevitably lead to death, *despite* the use of aggressive antibiotic therapy to prevent bacterial growth [12]. This is likely the result of high concentrations of bacterial toxins already accumulated in the body [13]. Therefore, to prevent LT-induced deaths, a detailed understanding of the pathogenesis is necessary. In vitro experiments indicated that LT may inhibit platelet function directly or indirectly [14], [15]. Among platelet-suppressive manifestations, thrombocytopenia commonly develops in anthrax patients and animal models [8], [14], [16]. Thrombocytopenia can be caused by two main reasons: a reduced production of platelets by the progenitor megakaryocytes [17], and an increased platelet loss by rapid consumption [18]. Previous studies have shown that PA binds to anthrax receptors in all lineages of hematopoietic progenitors in the bone marrow, including platelet precursor megakaryocytes [19], however, megakaryopoiesis suppression by LT has not been specifically characterized.

Megakaryopoiesis involves multipotential stem/progenitor cell commitment, nuclear polyploidization, cytoplasmic maturation, and the release of platelets [20]. The first stage of megakaryocyte development is the differentiation and proliferation of hematopoietic stem cells into bipotential erythroid/megakaryocytic cells and then into megakaryoblasts. The second stage involves nuclear polyploidization, demarcation membrane formation, cell size increase, and the expression of specific surface markers (CD41, CD61, or CD42b) [20], [21]. Megakaryocytes become polyploid cells through repeated DNA replication cycles without cytoplasmic division. The cell size correlates with the degree of polyploidization and maturation [22]. The final stage involves proplatelet formation and functional platelet release [23].

The ERK pathway is involved in the second stage of megakaryopoiesis in both thrombopoietin (TPO), a physiological positive regulator of megakaryopoiesis [24], and phorbol ester (12-*O*-tetradecanoylphorbol-13-acetate; TPA)-induced models [25], [26]. The inhibitory role of LT on MAPK pathways was shown to suppress cell survival of human endothelial cells and macrophages, and inhibit monocytic differentiation [27]–[29]. Based on these results, this study hypothesizes that LT may influence megakaryopoiesis by blocking differentiation or initiating the death of megakaryocytes. To test this hypothesis, in vitro models were used to investigate LT treatments in suppressing megakaryopoiesis; in vivo analyses in mice then evaluated LT-induced thrombocytopenia. This study also evaluated the protective effect of TPO in LT-challenged mice.

## Results

### LT Challenges Inhibited Human Megakaryocyte Colony Formation

**Figure 1:**
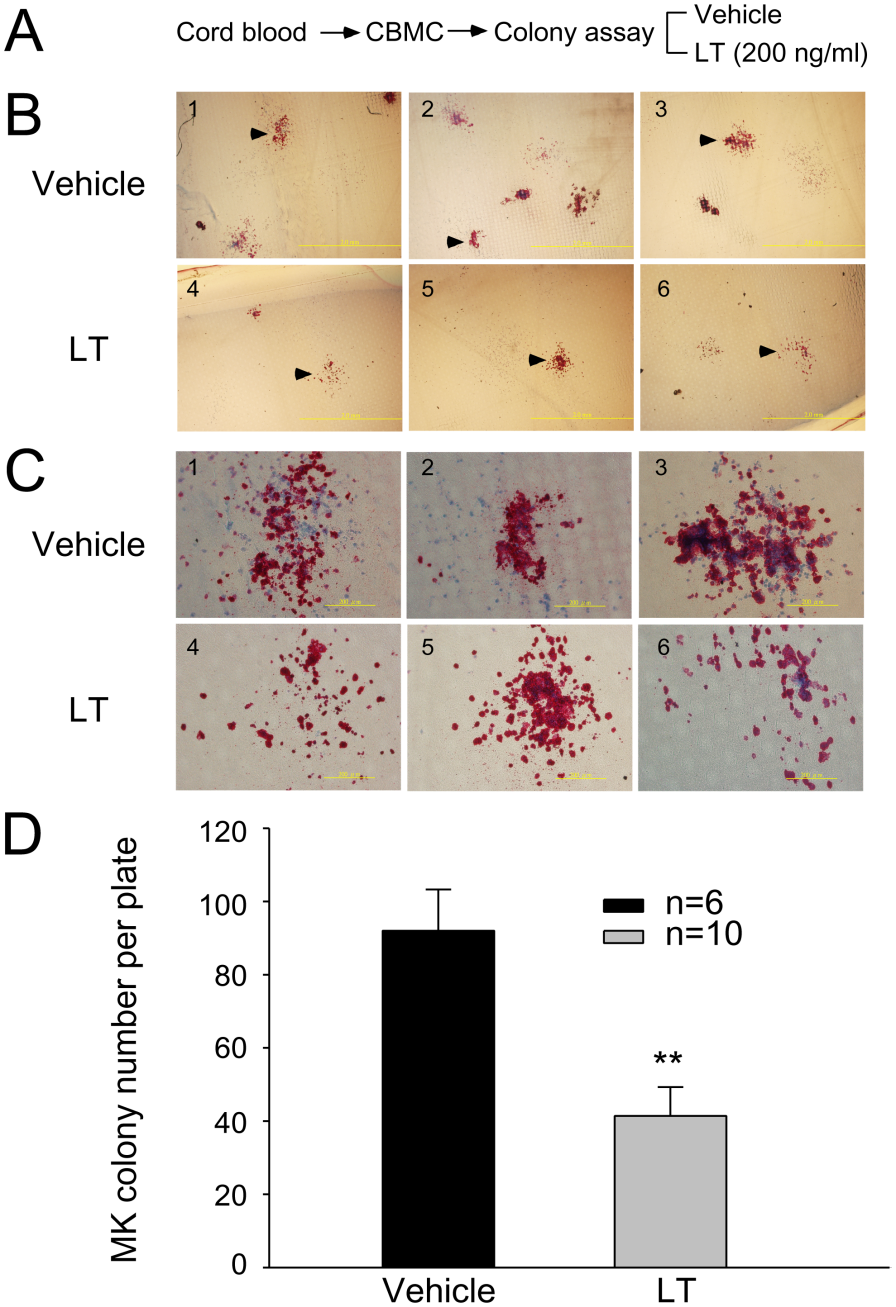
Suppressive effect of LT on CFU-MK formation. (A) The experimental outline. Human cord blood-derived mononuclear cells (CBMCs) were used in a megakaryocytic colony-forming unit (CFU-MK) assay. This figure shows the morphology (B–C) and quantified numbers (D) of megakaryocytic colonies. Arrowheads in the low magnification images (B-1 to B-6) indicate specific colonies that are highlighted in high-magnification photographs (C-1 to C-6) for each group (C). Scale bar: B, 2 mm; C, 200 µm. The cell-culture medium was used to dilute LT and was used as a treatment control in vehicle-groups. Data are representative of 3 independent experiments. One plate was seeded with 1.1×10^5^ cells. Data are reported as mean ± standard deviation (SD), **p<0.01 compared to vehicle groups.

To investigate whether LT interferes with megakaryopoiesis, a colony-forming unit-megakaryocytic (CFU-MK) assay was performed using human umbilical cord blood-derived mononuclear cells (CBMCs). Results revealed that LT challenge (200 ng/ml) reduced the number of megakaryocytic colonies significantly, compared to vehicle controls (Figure 1A, experiment outline; Figure 1B–C, representative images of CFU-MK; 1D, quantitative results, **p<0.01). In addition, the colonies in LT-treated groups consisted of relatively fewer and loosely distributed cells compared to the colonies in the vehicle groups (Figure 1B-C, vehicle vs LT). These results reveal the suppressive effect of LT on megakaryopoiesis in primary human progenitor cells.

### LT-mediated Suppression Leads to the Death of Premature/mature Megakaryocytes in a Model Using Human Cord Blood-derived CD34^+^ Cells

**Figure 2:**
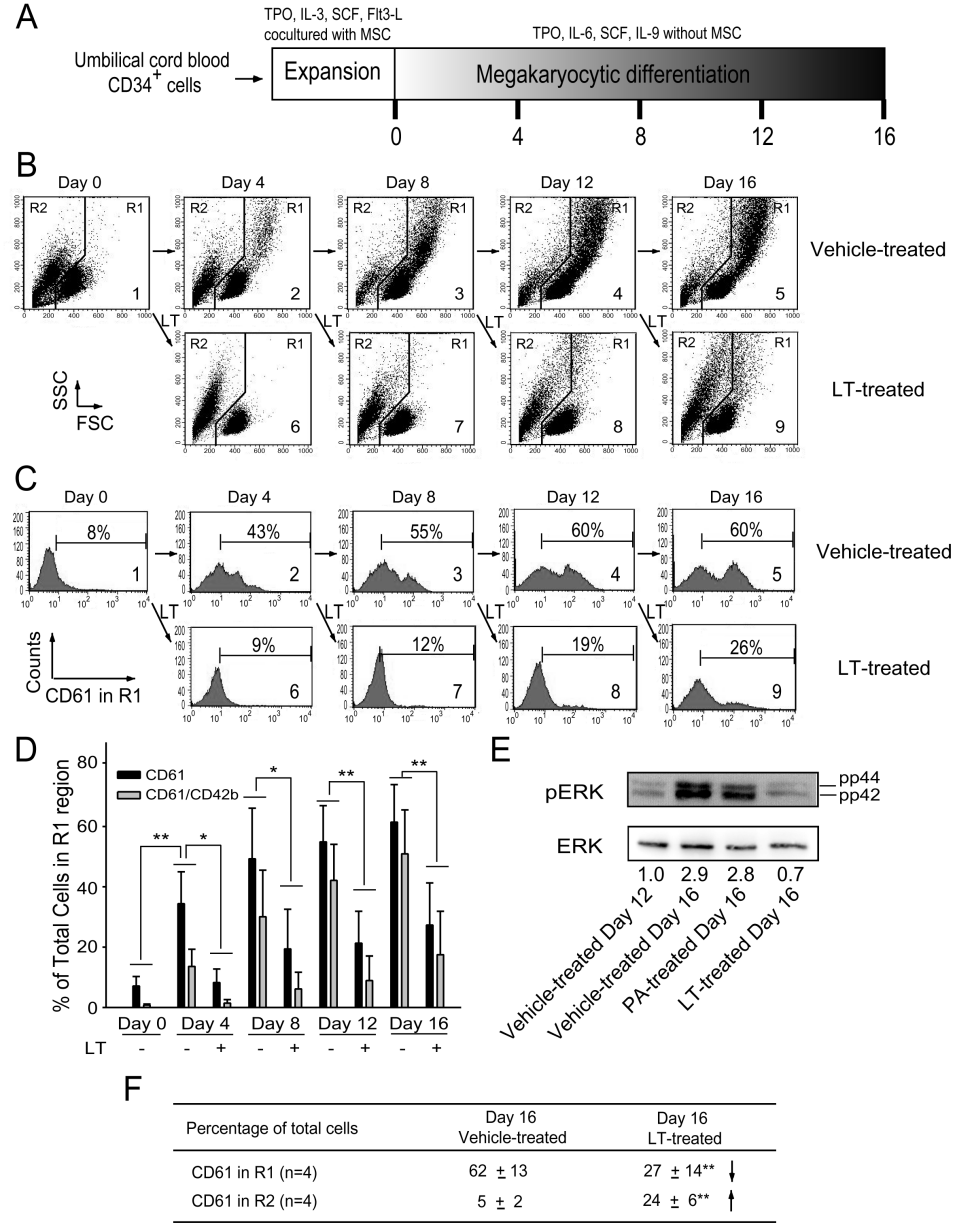
Suppressive effect of LT on in vitro megakaryocytic differentiation. (A) The experiment outline of in vitro megakaryocytic differentiation using human cord blood-derived CD34^+^ cells. Cells were firstly expanded on mesenchymal stem cell (MSC) monolayer and then subjected to a 16-day course of differentiation (0–16 day), in which LT and vehicle (diluents: cell-culture medium) were treated to various groups by day 0, 4, 8, and 12, respectively (B1–B9; C1–C9). Four days after LT treatments, megakaryocytic surface marker CD61 (GPIIIa; total megakaryocytes) and CD42b (GPIb; mature megakaryocytes) of each groups were then analyzed by flow cytometry on days 4, 8, 12, and 16, respectively. Flow cytometry analysis of the cell size (FSC) and cell granularity (SSC) at various time points are shown (B). The percentage of CD61^+^ cells in R1 regions (B) is illustrated in (C). Quantitative results on the percentage of CD61^+^ and CD61^+^/CD42b^+^ cells in R1 regions at different differentiation time points are indicated (D). The entire population of R1+ R2 cells was defined as 100%. Data are reported as mean ± standard deviation (SD) and represent 4 independent experiments. The images for Western blot of phosphorylated-ERK (pERK) and total ERK are shown (E). Relative gel intensities (fold change) after normalized with respective total ERK levels are indicated below the blot images, in which the Day 12 group was normalized to one fold. Summarized events occurred on day16 were shown (F). **p<0.01 compared to indicated groups.

**Figure 3:**
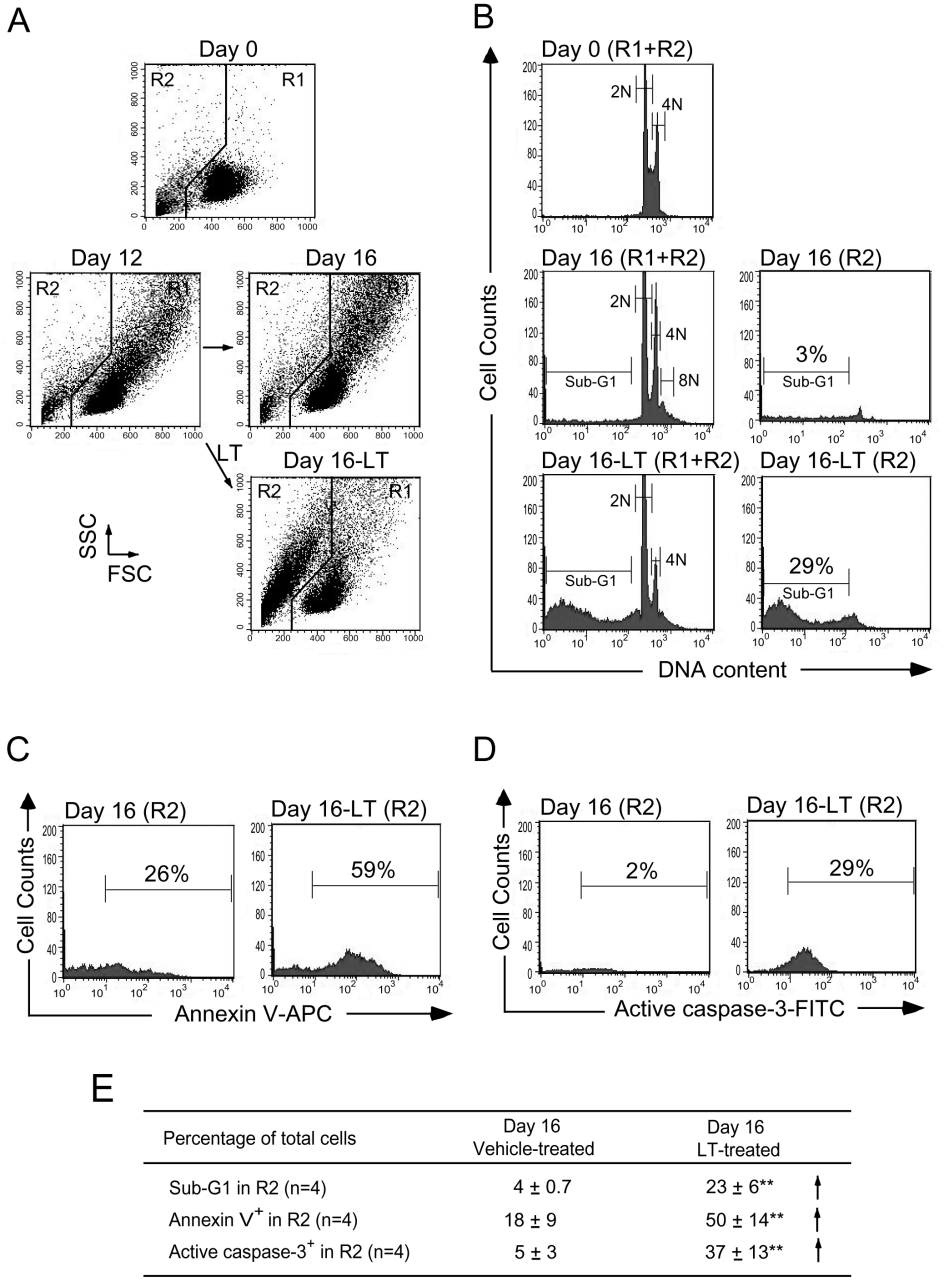
Characterizations of LT-induced hypoploid cells by apoptosis assay. The expanded human cord blood-derived CD34^+^ cells (Day 0) were treated with LT on day 12 and then analyzed on day 16 in aforementioned 16-day differentiation courses of megakaryocyte. The cell size (FSC) and cell granularity (SSC) are indicated (A). Propidium iodine (PI) staining revealed the cellular DNA contents, in which the sub-G1 hypoploid cells were increased in LT-treated groups (B). AnnexinV-APC (C) and active caspase-3 antibodies (D) were used to investigate the apoptotic changes of LT-treated cells by flow cytometry. Summarized events were shown on (E). Data are reported as mean ± standard deviation (SD) and represent 4 independent experiments. **p<0.01 compared to vehicle-treated groups.

**Figure 4:**
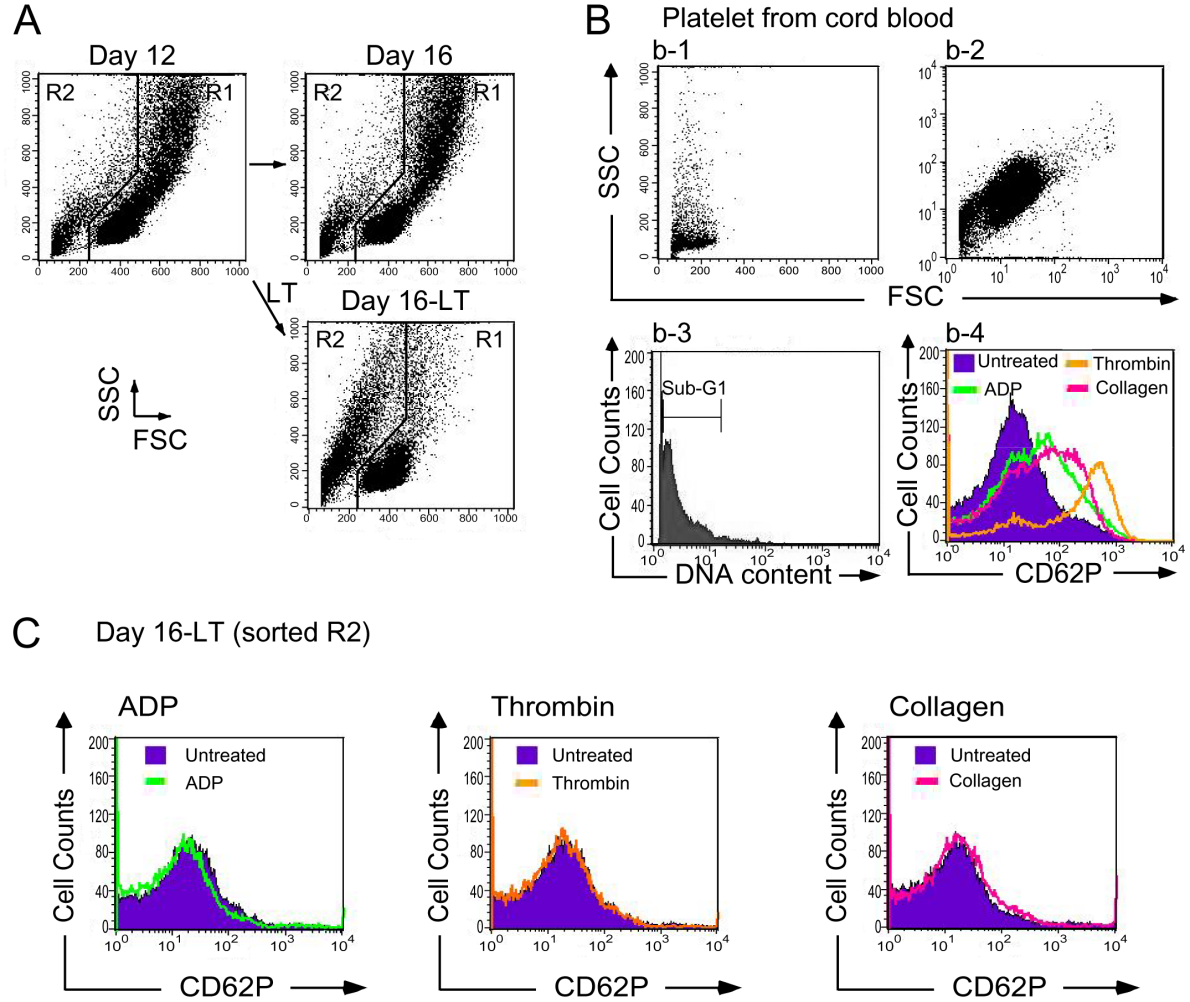
Characterizations of LT-induced hypoploid cells by platelet activation agonists. Human cord blood-derived CD34^+^ cells were treated with LT on day 12 and then analyzed on day 16 in aforementioned 16-day differentiation courses of megakaryocyte. The cell size (FSC) and cell granularity (SSC) are indicated (A). Size (FSC) and granularity (SSC) parameters of cord-blood platelets were plotted as linear scale (b-1) and logarithmic scale (b-2). The cellular DNA contents were revealed by propidium iodine (PI) staining (b-3). Agonist ADP, thrombin, and collagen-induced stimulation of the platelets (b-4) versus cell sorter-enriched R2-cells (day 16, LT-treated groups) (C) was revealed by enhanced surface staining of P-selectin (CD62P), a platelet activation marker. These results obtained from 3 independent experiments.

The inhibitory effect of LT may be divided into two categories, involving the inhibition of cell-differentiation and the inhibition of cell-survival/proliferation in megakaryocytes. In vitro megakaryocytic differentiation of cord blood-derived CD34^+^ hematopoietic stem cells (HSC) was analyzed over a 16-day course to verify the status (Figure 2A, 0–16 days). To investigate the inhibitory process in various stages, cells were divided into nine groups (Figure 2B1–9, 2C1–9), and then subjected to vehicle (diluents: cell culture medium; Figure 2B1–5, 2C1–5) and LT (Figure 2B6–9, 2C6–9) treatments at various time points (day 0, 4, 8, 12, and 16) after initiating the differentiation. One of these nine groups was treated with vehicle or LT at time points day 0, 4, 8, and 12, respectively (Figure 2B–C, groups 2–5 vehicle, groups 6–9 LT). The duration of toxin treatments was four days for each of the LT groups (Figure 2B–C, group 6: days 0–4, group 7: days 4–8, group 8: days 8–12, group 9: days 12–16). After the treatments, surface marker CD61 (GPIIIa; expressed on total megakaryocytes) and CD42b (GPIb; expressed on mature megakaryocytes) of each group were analyzed by flow cytometry (Figure 2B–D). Analyses revealed that the R1 cell-population of vehicle-treated control groups gradually experienced increases in cell size (FSC), cell granularity (SSC) (Figure 2B), the percentage of CD61^+^ (all megakaryocytes; Figure 2C and 2D), and the percentage of CD61^+^/CD42b^+^ (mature megakaryocytes; Figure 2D). In contrast, 4-day LT treatments caused a phenotypic change wherein the major population shifted from R1 (Figure 2B, larger cells) to R2 region (Figure 2B, smaller cells) at all stages, including the groups at day 4, 8, 12, and 16 (Figure 2B, groups 2–5 vs 6–9, respectively). The percentage of CD61^+^ (all megakaryocytes; Figure 2C, 2D, and 2F) and CD61^+^/CD42b^+^ cells (mature megakaryocytes; Figure 2D) in R1 region decreased after LT challenges. Western blot analysis was performed to investigate whether this suppression is associated with the blockage of MAPK pathway. Results show that ERK pathway was activated during the differentiation processes, while LT significantly suppressed such a response (Figure 2E). To coordinate various following analyses using same reference points to observe the effects of vehicle versus LT treatments on R1 and R2 cell populations, we placed representative figures that indicated the changes of cell size and granularity after the vehicle and/or LT treatments during differentiation-course day 12–16, consistently in Figure 2B (groups 4-to-5: vehicle effect; groups 4-to-9: LT effect), Figure 3A, and Figure 4A. Challenges of control protein PA to cultured cord blood CD34^+^ cells during megakaryocytic differentiation did not perturb the dynamic changes of cell size and granularity (Figure S1A), surface markers CD61 and CD42b expression (Figure S1B and unpublished results), and ERK activation (Figure 2E). Because dead cells are usually smaller than normal cells (R2, Figure 3A), propidium iodine (PI), Annexin V, and antibodies against active caspse-3 were used to verify whether these cells were entering the sub-G1 phase (Figure 3B) and apoptosis is involved (Figure 3C–3E), and group 4, 5, and 9 were used as representative. Flow cytometry analysis revealed that the percentage of hypoploid cells (DNA content less than 2N, sub-G1) in R2 region was higher in LT-challenged groups (Figure 3A, cell size and granularity; Figure 3B, DNA content). In addition, apoptosis is likely involved in LT-mediated cellular changes as compared with PA controls (Figure 3C–3E and Figure S1C–1E). Since platelet biogenesis also involves apoptotic processes [30],[31], and the R2-population contained a high percentage staining patterns of platelet-marker CD61 (68% CD61^+^ in the R2 region, unpublished results), these R2 cells may still possible be platelet-like fragments instead of death cells. Platelets isolated from human cord blood were used as normal controls to verify this conjecture. Although the DNA contents of R2 cells are similar to platelets (Figure 3B vs. Figure 4B, b-3; sub-G1), flow cytometry data revealed that LT-challenged R2 cells did not fully match the size and granularity properties of mature platelets isolated from cord blood (Figure 4A, R2 vs Figure 4B, b-1 showed as a linear scale, b-2 showed as logarithmic scale). Induction of surface P-selectin (CD62P), a platelet activation marker, is a method for verifying the functional properties of platelets [32]–[34]. Data revealed that platelet-agonists including adenosine diphosphate (ADP), thrombin, and collagen failed to induce equivalent expression levels of surface P-selectin on those R2-region cells compared to cord blood platelets (Figure 4C, vs 4B, b-4). These results suggest that the small, hypoploid CD61^+^ populations in LT-challenged groups were likely fragments of dead megakaryocytic cells, rather than functional platelets. These results suggest that LT suppresses megakaryopoiesis by initiating cell death of premature and mature megakaryocytes.

### LT-mediated Suppression Leads to Cell Death of Megakaryocytes in a Model Using Mouse Bone Marrow Mononuclear Cells

**Figure 5:**
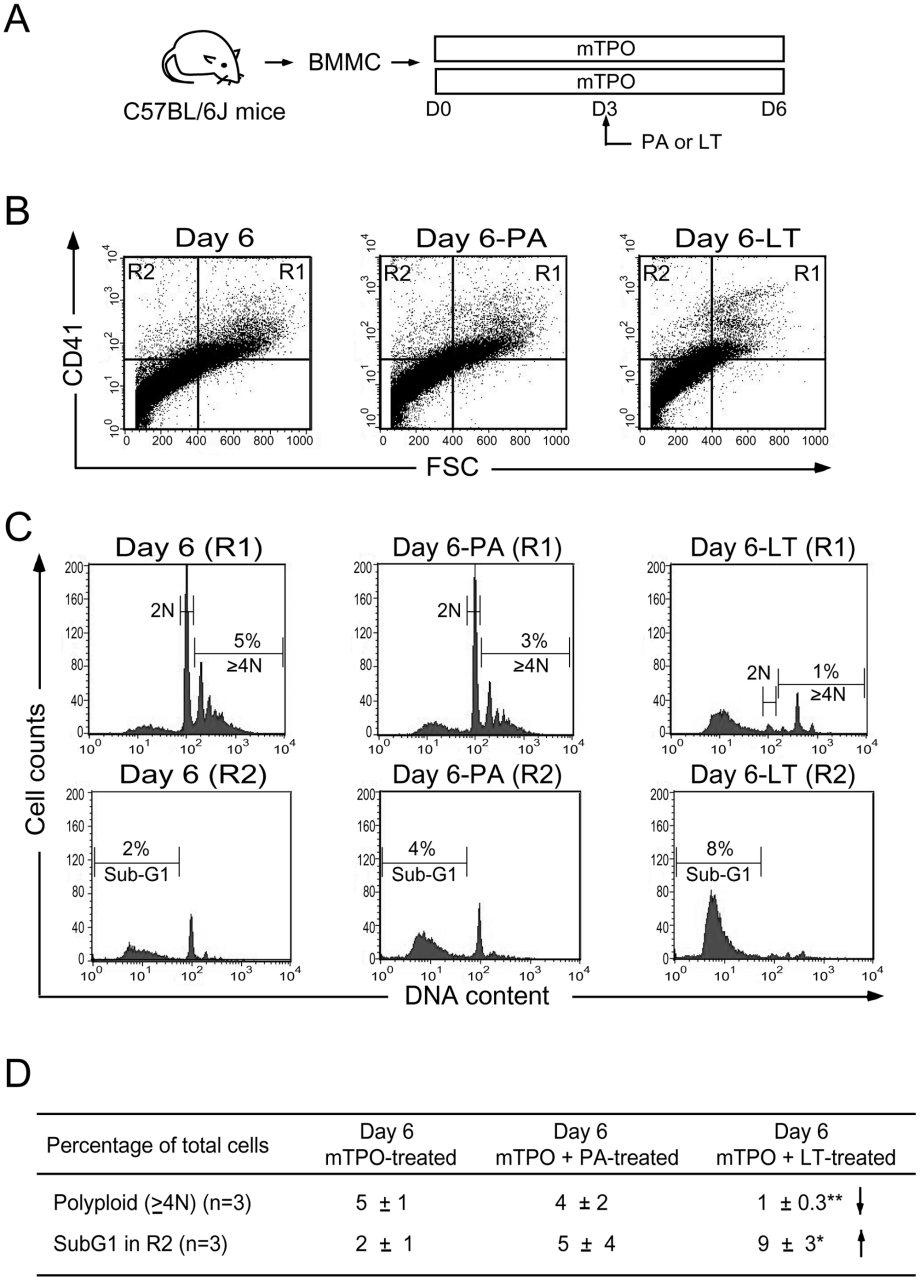
Suppressive effect of LT on mouse bone marrow cells. (A) The experimental outline. Mononuclear cells were isolated from bone marrow (BMMCs) and induced to become megakaryocytes in the presence of mTPO in a 6-day course. The cell size (FSC), megakaryocytic surface marker (CD41) (B) and DNA content (C) were analyzed by flow cytometry. Summarized events were shown on (D). Data are reported as mean ± standard deviation (SD) and represent 3 independent experiments. *p<0.05, **p<0.01 compared to mTPO-treated groups.

Before the in vivo analyses using a mouse model, we would like to investigate whether the behavior of murine megakaryocytes is similar to human cells. Megakaryocytic differentiation of mouse bone marrow-derived mononuclear cells (BMMCs) was initiated in vitro by supplements with mouse TPO (mTPO). The number of mouse BMMCs carrying megakaryocytic features (CD41^+^ and polyploid cells) was increased and could be detected at the end of the assay (Figure 5A, experiment outline, Figure 5B, R1 region and Figure 5C, Day 6 groups). By contrast, LT challenges suppressed the induction of size and polyploidy of these CD41^+^ cells (Figure 5B and 5C, Day 6-LT groups).

### Thrombopoietin Treatments Reduced LT-mediated Mortality, Thrombocytopenia, And Megakaryopoiesis Suppression in Mice

**Figure 6:**
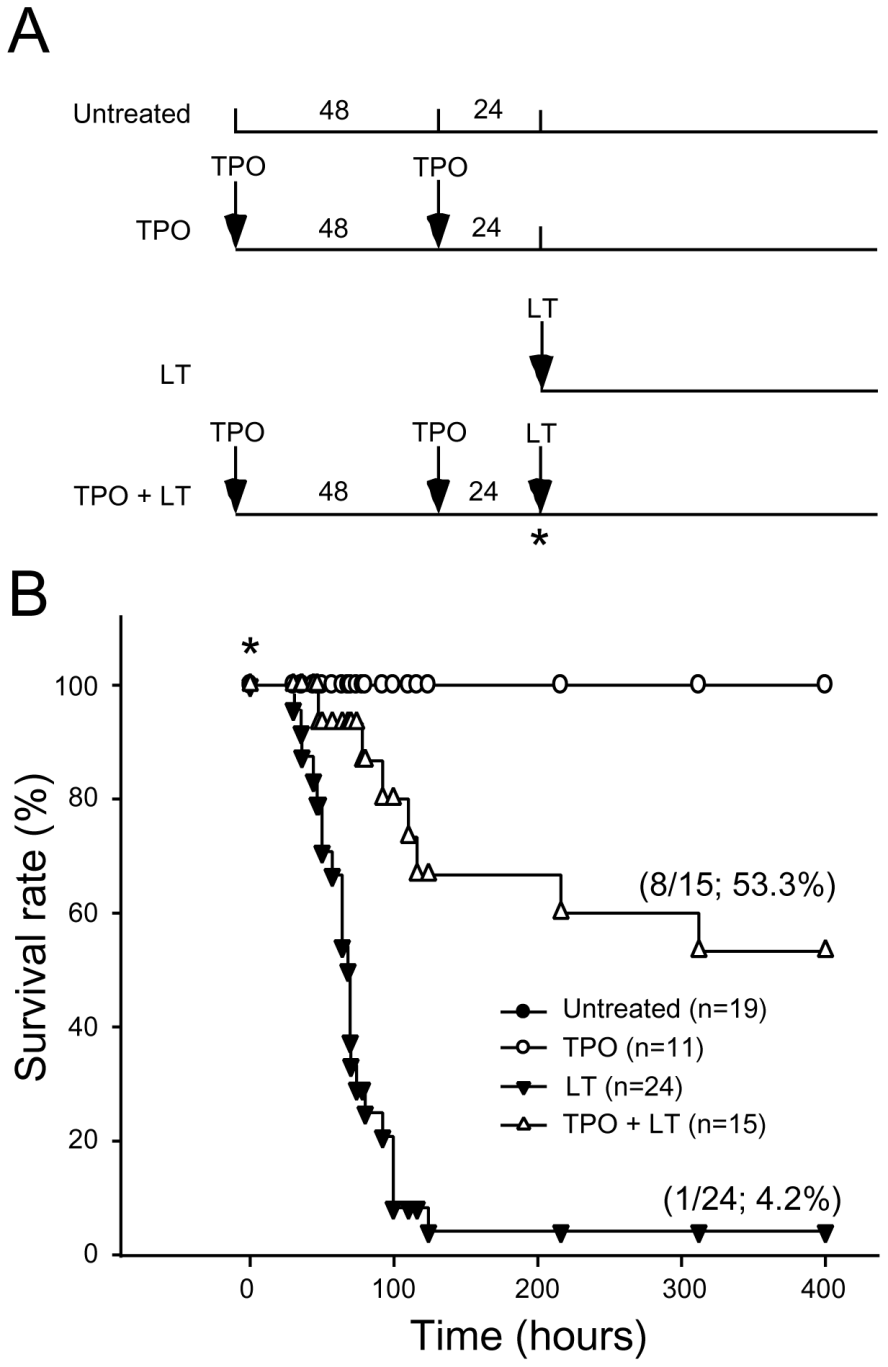
TPO treatments reduced LT-mediated mortality in mice. (A) The experimental outline. (B) The survival rates of mice treated with TPO, LT, or TPO plus LT are shown. Untreated mice were used as negative controls. Asterisk (*) marks in (A) and (B) indicate the starting time point for recording survival rate.

**Figure 7:**
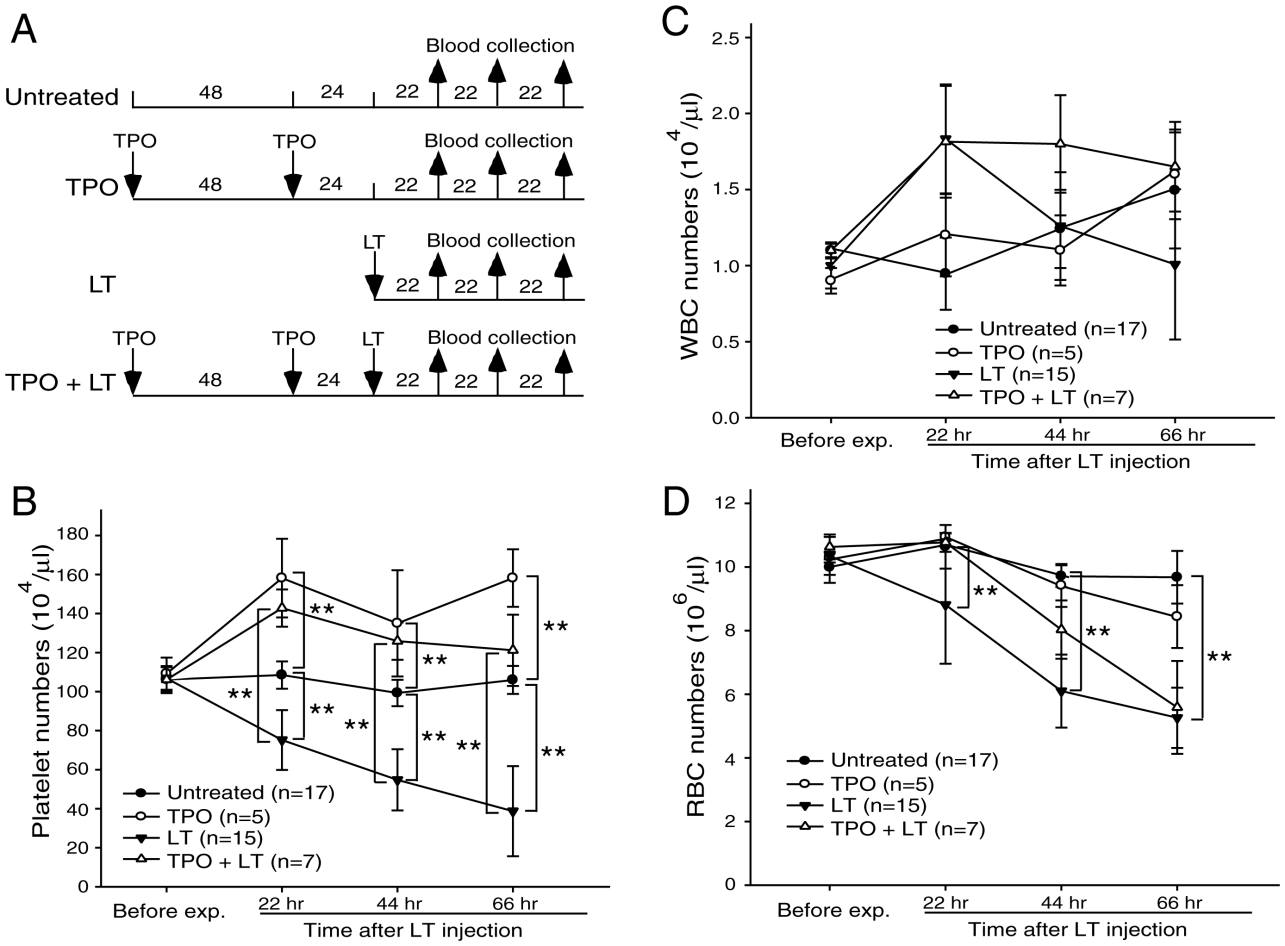
TPO pretreatments ameliorated LT-mediated thrombocytopenia in mice. (A) The experiment outline used in measurement of hematopoietic parameters. (B–D) The platelet, WBC, and RBC counts of mice treated with TPO, LT, or TPO plus LT at 22, 44, and 66 hours, respectively. The measurements of platelet, WBC, and RBC counts right before TPO and/or LT treatments [prior to first TPO treatments of TPO groups in (A)] were served as basal levels (Before exp. groups, C–D). Untreated mice were used as negative controls. **p<0.01 compared between indicated groups. Data are reported as mean ± standard deviation (SD) and representative of 2 independent experiments.

**Figure 8:**
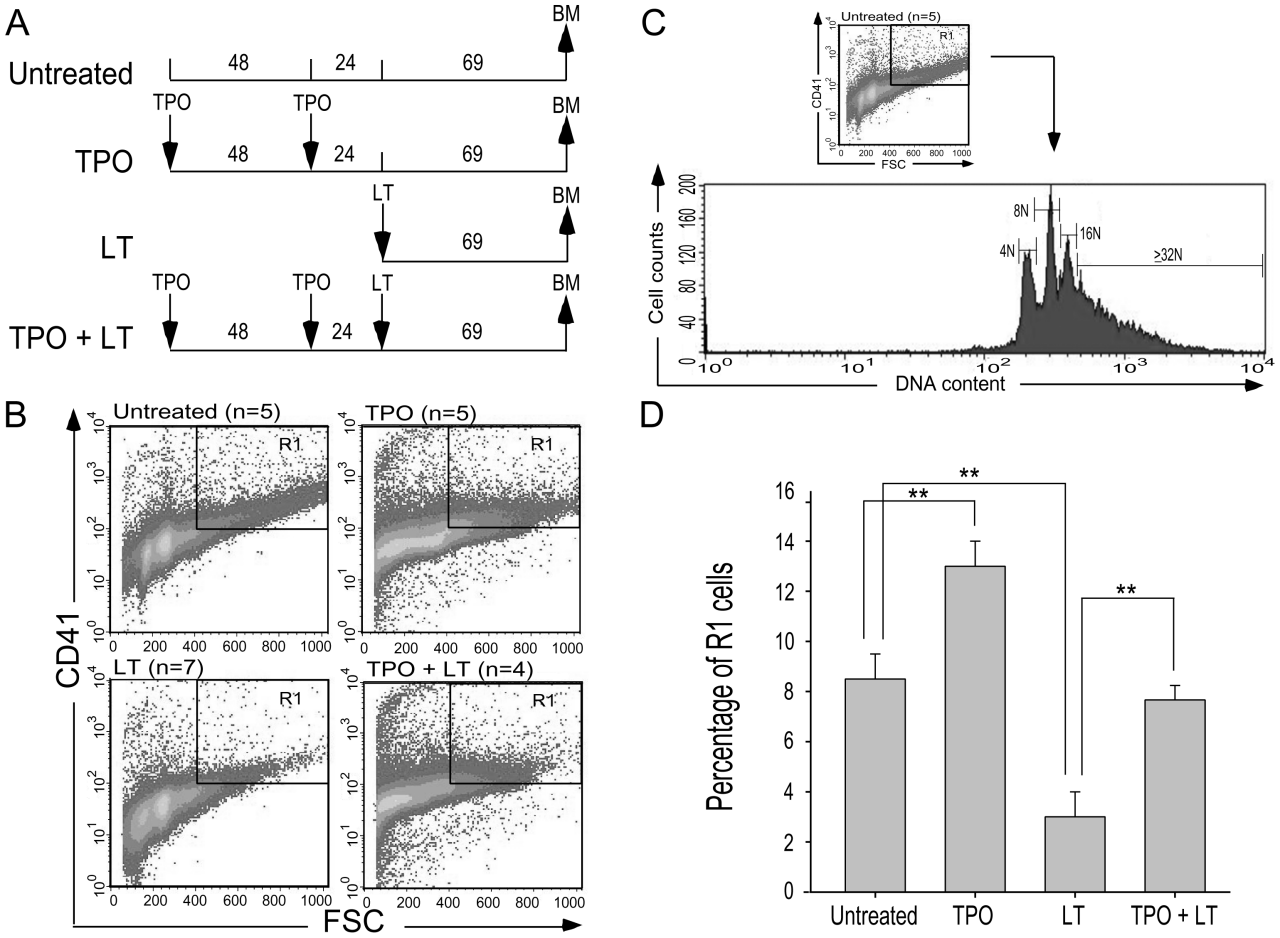
TPO pretreatments ameliorated LT-mediated megakaryopoiesis suppression in mice. (A) The experiment outline used in bone marrow (BM) experiments. Flow cytometry analysis using mouse bone marrow cells was performed at 69 hours after LT treatments. Following a previously described method [69], the population of polyploid megakaryocytes was gated as CD41 high (fluorescent intensity>10^2^) and large (FSC>400) cells in all groups (B, R1 regions). The DNA content of R1 cells in the untreated group was illustrated (C), and relative R1 cell population (percentage of total 2×10^5^ cells) was quantified (D). **p<0.01 compared between indicated groups.

If thrombocytopenia and megakaryocytic suppression indeed play certain roles in LT-mediated pathogenesis, theoretically, we might provoke megakaryopoiesis to overcome such adverse effect. Thrombopoietin (TPO), also known as megakaryocyte growth and development factor, is a native cytokine responsible for enhancing megakaryocytic development [24]. Experiments on mice showed that a single injection of a lethal dose of LT (1.5 mg/kg) induced high mortality within 124 hours (survival rate 4.2%; 1/24) (Figure 6A, experiment outline; Figure 6B, LT groups), whereas no lethality occurred in the control groups injected with PA (unpublished data). In contrast, TPO pretreatment delayed the onset and reduced the percentage of lethality (Figure 6B, survival rate 53.3%, 8/15). All surviving mice remained alive for subsequent 2 months (unpublished data). To determine whether reduced mortality was associated with an ameliorated thrombocytopenic response, complete blood cell counts of TPO-treated mice were measured in parallel at 22, 44, and 66 hours post-LT challenge (Figure 7A, experiment outline). Analysis results indicate that challenge of LT, but not PA, significantly reduced the platelet counts in mice (Figure 7B, untreated vs LT groups; Figure S2, PA groups). Pretreatment of TPO significantly increased the level of circulating platelets and reduced LT-induced thrombocytopenic responses (Figure 7B, untreated vs TPO; LT vs TPO+LT groups, **p<0.01). By contrast, pretreatments of TPO did not increase circulating white blood cells (WBCs) and red blood cells (RBCs) (Figure 7C and 7D, untreated vs TPO). To verify whether TPO treatment ameliorates LT-suppressed megakaryopoiesis in vivo, the percentage of CD41^+^ polyploid megakaryocytes (cells gated with FSC>400 and CD41>10^2^, DNA contents ≧ 4N) in mice bone marrow cells were examined (Figure 8A, experiment outline, 8B–8D). At 69 hours post-lethal dose treatment of LT (1.5 mg/kg), the percentage of mouse bone marrow CD41^+^ polyploid cells dropped significantly (Figure 8B and 8D, untreated vs LT groups, **p<0.01). These results demonstrate the suppressive role of LT on megakaryopoiesis in vivo, which agrees with in vitro human CFU-MK assay (Figure 1), and in vitro differentiation assays using human CBMCs and mouse BMMCs (Figure 2–5). At the same time, TPO pretreatments ameliorated LT-mediated reduction of polyploid megakaryocytes significantly (Figure 8B and 8D, LT vs TPO+LT groups). These results reveal that TPO-amelioration on LT-mediated megakaryocytopoiesis suppression is associated with reduced mortality and thrombocytopenia in toxin-challenged mice.

## Discussion

Evidence from this study suggests that the megakaryocyte is one of the target cells of LT, and the suppression of megakaryopoiesis is one of the mechanisms of LT induced thrombocytopenia. This study employs in vitro megakaryopoiesis models using primary human cord blood-derived CD34^+^ stem cells and mouse bone marrow cells to analyze the suppressive effects of LT. Results show that LT treatments restricted the expansion of CD61^+^ (total) and CD61^+^/CD42b^+^ (mature) megakaryocytes (Figure 2B, R1 regions), and substantially resulted smaller phenotype of CD61^+^ and CD61^+^/CD42b^+^ cell populations (Figure 2B, R2 regions and Figure 2F, unpublished results). An intriguing question is whether these R2-CD61^+^ signals are revealing platelets or debris of dead-cell. Implicated by PI (Figure 3B, sub-G1), Annexin V (Figure 3C), active caspase-3 antibody staining (Figure 3D), and platelet-activation (Figure 4C), analyses data reveal that these R2 signals were likely hypoploid apoptotic cells rather than platelets, because they lacked functional platelet responses (Figure 4C). These results suggest that the suppression of megakaryocytes is in part mediated by the killing of various megakaryocytic subpopulations (Figure 2, total: CD61^+^; mature: CD61^+^/CD42b^+^). This agrees with the suppressive effects of LT in the CFU-MK assay (Figure 1), in megakaryopoiesis in bone marrow (Figure 8), and in the death of murine megakaryocytes (Figure 5). The cord blood cell model further indicates that LT induces megakaryocytic apoptosis, which is associated with ERK suppression (Figure 2E). These results collectively suggest that LT suppresses megakaryopoiesis by suppressing the survival of megakaryocytes.

Thrombocytopenia is a common manifestation in anthrax patients and animal models [8],[14],[16]. Experimentally induced thrombocytopenia might not always lead to hemorrhage in mice (e.g., thrombocytopenia through anti-platelet Ig treatments [35] and authors’ unpublished results), unless these mice are suffering from coagulopathy [33] and/or vascular lesions [36],[37]. Severe coagulopathy disseminated intravascular coagulopathy and vascular lesions are associated with thrombocytopenic responses, and studies have suggested that they may be part of the pathophysiology of anthrax patients and animal models [16],[38]–[42]. Specifically, LT induces TNF-α independent hypoxia [8]. Hypoxia can cause platelet consumption, an elevated D-dimer, and coagulopathy in many other diseases [43]. In some hypoxic circumstances, however, platelet consumption is not always associated with D-dimer elicitation [43],[44]. In this study, we found that LT does not elicit a significant elevation of plasma D-dimer (48, 72, and 96 hours post LT treatments, unpublished results), which indicates that coagulopathy is likely not involved. Anthrax LT might impair platelet functions indirectly [15], whereas hypoxic conditions can alter platelet surface protein expression and induce procoagulant behavior [43]. Therefore, LT-induced platelet count reduction may be partially mediated through a coagulopathy-independent enhancement of platelet clearance. The mechanism, however, must be further clarified. This study also suggests that megakaryocytic suppression is one of the mechanisms of LT in inducing thrombocytopenia. Platelet survival is 3.5–4.5 days in rodents versus 7–10 days in humans [45],[46]. Thus, platelet reduction is estimated at 22–29% per day if new platelet supplies are blocked in mice. This matches our platelet count data, which showed an approximately 15–30% reduction over each 22-hour interval (Figure 7B). In addition, approximately 2–4 days are required to initiate mortality in LT-challenged mice (Figure 6B, LT groups). Thus, megakaryocytic suppression and exacerbated thrombocytopenia are likely associated with the onset of mortality (Figure 6–8). The ameliorative effect of TPO in the LT mouse model may further support this association.

Thrombopoietin TPO, the ligand for the c-Mpl receptor, is the principal physiological regulator of megakaryopoiesis and thrombopoiesis, and acts primarily on the proliferation and maturation of megakaryocytes [24]. As TPO may up-regulate platelet counts to counteract thrombocytopenia, its protective effect during LT challenge was evaluated. The administration of TPO reduced both the severity and the duration of LT-induced thrombocytopenia (Figure 7–8), and it increased the survival rate of LT-injected mice (Figure 6). Since TPO is also critical for maintaining certain hematopoietic stem cells in addition to megakaryocytes [47],[48], its protective effect on LT-induced lethality may theoretically also involve other mechanisms independent of megakaryopoiesis. However, TPO treatments did not significantly increase circulating WBCs and RBCs in the control and LT-challenged groups (Figure 7C and 7D). This suggests that the up-regulating effect of TPO on hematopoietic cells is primarily focused on megakaryocytes and platelets. From a clinical perspective, TPO treatment after infection is more feasible than TPO-pretreatment. Unfortunately, post-treatments of TPO after LT injection only prolonged survival time (Figure S3, LT: 77–101 hours vs LT+TPO: 90–185 hours, *p* = 0.0367), and did not increase the survival rate of LT-injected mice. Platelet generation from megakaryocytes requires 5 days in humans [49], whereas the process requires a much shorter process of 2–3 days in rodents [50]. Despite this short time period, megakaryocytes may not mature in time during the post-treatments (50% mortality occurred at 144 hr post LT treatments, Figure S3B, LT+TPO groups). The time course for TPO-induced megakaryopoiesis may explain why pretreatments of TPO increased the survival rate, whereas post-treatments of TPO only prolonged the survival period (Figure 6B vs Figure S3B).

The time course required by LT to elicit lethality in the experimental mice was shorter than that required by actual *B. anthracis* infections in patients (mice 3–5 days vs patients 5–8 days [51],[52]). This increases the possibility that thrombopoiesis-promoting agents such as TPO can still induce megakaryopoiesis before the onset of lethality and thus can be applied therapeutically in clinical settings. High concentrations of LT accumulated in the body [13] can lead to unavoidable death even after aggressive antibiotic therapy [12]. Because TPO is beneficial to LT-injected mice, TPO treatment can theoretically complement other supporting or independent treatments (e.g., antibiotics) for patients. Despite early successes in treating thrombocytopenic patients, clinical trials of recombinant human TPO were discontinued after autoantibodies against TPO were elicited, and an associated drop of platelet counter was observed, in a small group of healthy volunteers [53]. To avoid this side effect, second-generation TPO-receptor agonists, such as romiplostim and eltrombopag, can be used as feasible alternatives to clarify the potential therapeutic approach.

In summary, this study is the first to demonstrate that suppression of megakaryopoiesis is involved in LT-mediated thrombocytopenia. This study also shows that TPO treatment has protective effects. Because specific clinical treatments to overcome LT-mediated pathogenesis are still lacking, these findings might help researchers identify feasible approaches for treating anthrax.

## Materials and Methods

### Ethics Statement

Cord blood and umbilical cord samples from full-term pregnancies were collected by Mennonite Christian Hospital and the Buddhist Tzu Chi Stem Cell Center, Hualien, Taiwan. Informed written consents were provided by participants and obtained using protocols approved by the Research Ethics Committee of Mennonite Christian Hospital (approval ID: 09-12-046-ER and 09-12-047-ER) and Buddhist Tzu-Chi General Hospital (approval ID: IRB097-14). All human samples were anonymized. The research methods involving the experimental mice were in accordance with the national guidelines of Animal Protection Act (Taiwan) and approved by the Institutional Animal Care and Use Committee, Tzu Chi University (approval ID: 97060; Project: Molecular characterization of megakaryocytic differentiation).

### Toxins

*B. anthracis* (ATCC 14186)-derived lethal toxin (LT) and PA were purified as previously described [54],[54,55]. Doses of LT were 1∶5 amounts of LF and PA (i.e., 120 µg LT consists of 20 µg LF plus 100 µg PA).

### Megakaryocytic Colony Forming Unit (CFU-MK) Assay

To quantify human cord blood-derived megakaryocytic progenitors, CFU-MK assay was performed following the manufacturer’s instructions (MegaCult-C, StemCell Technologies, Vancouver, Canada). Ficoll-Paque Plus (GE Healthcare Bio-Sciences, Piscataway, NJ) was used to prepare purified mononuclear cells from cord blood. These mononuclear cells (1.1×10^5^) were seeded in a double chamber culture slide with serum-free Iscove’s Modified Dulbecco’s Medium (IMDM) containing cytokines. These cells were treated with or without LT (200 ng/ml) during the experiment, in which the cell culture medium was used as a diluent for LT and was served as a vehicle control. After incubation at 37°C with 5% CO_2_ for 12 days, culture slides were fixed, stained by anti-GPIIb/IIIa antibodies and counterstained with 1% (w/v) Evans Blue to obtain cell images and quantitative results of MK colonies. All experiments were performed in duplicate or triplicate and repeated at least 3 times.

### Megakaryocyte in vitro Culture and Flow Cytometry Assay

#### Human cord blood

Following the manufacturer’s instructions, human CD34^+^ cells were isolated from cord blood mononuclear cells using a CD34 MicroBead kit (Miltenyi Biotec, Bergisch Bladbach, Germany). To expand the cell number, CD34^+^ cells were cocultured with mesenchymal stem cells (MSC) from Wharton’s jelly of umbilical cords. At this stage, cells were cultured in IMDM (Gibco) containing 10% fetal bovine serum (FBS) (Biological Industries, Kibbutz Beit Haemek, Israel), 10 ng/ml recombinant human (rh) TPO (PeproTech), 20 ng/ml rh IL-3 (PeproTech), 30.5 ng/ml rh stem cell factor (SCF) (PeproTech), and 22.3 ng/ml rh Flt-3 ligand (FL) (PeproTech) for 4–5 days [56],[57]. To induce megakaryocytic differentiation, CD34^+^ cells (5×10^5^ in one 6-well dish) were cultured at 37°C in 5% CO_2_ and 100% humidity without MSC, using differentiation medium-IMDM supplemented with 3% FBS, 2 mM l-glutamine, 100 U/l penicillin, 100 mg/ml streptomycin, and cytokines including 50 ng/ml rh TPO, 7.5 ng/ml rh IL-6 (PeproTech), 1 ng/ml rh SCF, and 13.5 ng/ml interleukin 9 (IL-9) (PeproTech) [58]. To sustain the differentiation for 16 days, differentiation medium was freshly prepared and renewed every 4 days. Cells were divided into nine groups. LT (10 ng/ml) and vehicle (culture medium) were added to the medium at various differentiation stages by days 0, 4, 8, and 12, respectively. After 4-day toxin treatments, each group of cells was analyzed by flow cytometry. The levels of megakaryocytic specific surface markers were determined by fluorescein-isothiocyanate (FITC)-conjugated anti-human CD61 (eBioScience, San Diego, CA) and allophycocyanin (APC)-conjugated anti-human CD42b (Biolegend, San Diego, CA) antibodies. To analyze apoptotic cells, Annexin V-APC (BD Pharmingen, CA, USA) and FITC-conjugated anti-active caspase-3 (BD Pharmingen) antibodies were used. Digitized results were then analyzed and quantified using the CellQuest program (Becton-Dickinson).

#### Mouse bone marrow

Bone marrow cells were isolated from C57BL/6J mice (male, 6–8 weeks old) by flushing femurs and tibiae with serum-free Dulbecco’s Modified Eagle’s Medium (DMEM) through 30-gauge needles. After depleting red blood cells by ACK buffer (0.15 M NH_4_Cl, 10 mM KHCO_3_, 0.1 mM Na_2_EDTA, pH 7.2–7.4) for 10 minutes at 4°C, cells were cultured (1×10^6^ in one 6-well dish) at 37°C in 5% CO_2_ and 100% humidity using differentiation medium-DMEM supplemented with 10% FBS, 2 mM l-glutamine, 100 U/l penicillin, 100 mg/ml streptomycin, and 50 ng/ml rmTPO (PeproTech) [59],[60]. Megakaryocytic specific surface markers were determined by fluorescein-isothiocyanate (FITC)-conjugated anti-mouse CD41 (BD Pharmingen) antibodies.

### DNA Content Analysis

After washes (phosphate buffered saline: PBS), fixation (ice-cooled 70% ethanol in PBS at –20°C for 2 hours), and additional washes (PBS), cells were resuspended in 500 µl staining solution containing propidium iodine (PI) (20 µg/ml PI, 0.1% Triton X-100 and 0.2 mg/ml RNase A in PBS), and incubated at 25°C for 30 minutes. The DNA content of cells was analyzed using a flow cytometer (FACSCalibur, Becton-Dickinson, San Jose, CA, USA).

### Platelet Isolation

Platelets were isolated from human cord blood as described [14]. The samples were centrifuged at 120×g for 5 minutes at 25°C, and washed with Tyrode’s solution. Purified platelets and cell sorter (BD FACSAria™ II)-isolated cells (R2 region) were stimulated with 40 µM adenosine diphosphate (ADP) (Helena Laboratories), 0.1 U/ml thrombin (Sigma-Aldrich), and 10 mg/l collagen (Sigma-Aldrich) for 30 minutes at 25°C without stirring. These platelets were then stained with phycoerythrin (PE)-conjugated anti-human/mouse P-selectin (CD62P) (eBioScience, San Diego, CA) antibodies (1∶100) for 30 minutes at 25°C and analyzed by flow cytometry (FACSCalibur, Becton-Dickinson, San Jose, CA, USA).

### Western Blotting

Western blotting was performed as described previously [14],[61]. The protein of cell extracts (20 µg protein/lane) was separated by 8% SDS-PAGE and then transferred to nitrocellulose membranes (Hybond-C Extra, Amersham Bioscience, Little Chalfont, UK). The membranes were incubated with a protein-blocking buffer [5% skim milk and 0.2% Triton X-100 in TBS (1.5 M NaCl, 0.2 M Tris–HCl, pH 7.4)] for 1 hour at 25°C, and then probed with antibodies against phosphorylated-ERK (pERK, Cell Signaling Technology, Beverly, MA, USA) and ERK (BD Transduction Laboratories). Cellular ERK levels were served as internal and loading controls. The intensities of the blots were developed and quantified by Immobilon Western Chemiluminescent HRP Substrates (Millipore, Billerica, MA, USA) and a Biospectrum AC System (UVP, Upland, CA, USA), respectively.

### TPO Treatments on the Reduction of LT-induced Mortality

C57BL/6J mice (males, 8–10 weeks old) were purchased from the National Laboratory Animal Center (Taipei, Taiwan) and maintained in a specific pathogen-free condition in the experimental animal center of Tzu Chi University. As retro-orbital injection is an accepted intravenous route for administration of testing compounds [62], both recombinant murine TPO (rmTPO, CytoLab/PeproTech Asia, Rehovot, Israel) [63] and LT were administrated into C57BL/6J mice using this method. This rmTPO is expressed and purified from *E. coli* in an intact form without glycosylation or PEGylation. Mice were treated with rmTPO (CytoLab/PeproTech Asia, Rehovot, Israel) (0.25 µg/mouse, in 250 µl saline) twice at 72 and 24 hours prior to injection of a lethal dose LT (1.5 mg/kg in 250 µl saline, retro-orbital injection) to investigate the protective effect. The TPO dose (0.25 µg/mouse) was determined based on previous studies [63]–[66]. Three TPO doses (0.25 µg, 0.5 µg and 1 µg per mice) were tested on the amelioration of LT-mediated pathogenesis, in which 0.25 µg/mouse got a best rescue effect. The dose of LT (1.5 mg/kg) was determined according to previous literatures [8],[14] [67]. According to estimation of LT-turnover in vivo [54], this dose of LT is within the clinical range detected in patient [68]. Mice treated with (TPO groups) or without TPO (untreated groups) that lacked further LT injections were served as control groups. The mortality and survival time of mice were recorded after the LT treatments. To measure the hematopoietic parameters of mice, 50 µl blood samples were collected 22, 44, and 66 hours after LT treatments from retro-orbital sinus/plexus, and then subsequently mixed with 450 µl anticoagulant citrate dextrose (ACD)-containing diluents (1∶9) [35]. The hematopoietic parameters were determined using an automated hematology analyzer (KX-21, Sysmex Corporation, Kobe, Japan).

### DNA Content Analysis of Megakaryocytes in Bone Marrow

C57BL/6J mice were retro-orbitally injected with LT and recombinant murine TPO and as described in the methods section (**TPO treatments on the reduction of LT-induced mortality**). After the retro-orbital injection of a lethal dose LT (1.5 mg/kg in 250 µl saline) for 69 hours, mouse bone marrow cells were collected by flushing femurs and tibiae with ice-cold MK-buffer/3% BSA (Ca^2+^/Mg^2+^-free PBS containing 3% BSA; 2.8 µM prostaglandin E1; 5.5 mM D-glucose; 10.2 mM trisodium citrate, pH 7.3) supplemented with anticoagulant ACD (at 4∶1 vol/vol) through 30-gauge needles. Megakaryocytes derived from bone marrow were enriched and analyzed as previously described [69]. The cell suspensions were passed through a 55 µm nylon mesh to remove cell aggregates. Aliquots of 5 ml bone marrow suspension were layered over 5 ml of Percoll (density = 1.06 g/ml) (Sigma-Aldrich) and overlaid with 3 ml of MK buffer/0.3% BSA, then centrifuged at 400×g for 20 minutes at 25°C. The cells in the middle layer and the middle layer-Percoll interfaces were then collected. These cells were resuspended in ice-cold MK buffer/3% BSA and centrifuged at 300×g for 10 minutes at 4°C. After washes, cells (1×10^7^) were incubated in 100 µl MK buffer/0.3% BSA with anti-mouse CD41-FITC antibody (BD) for 1 hour at 37°C. After PBS washes, fixation (70% ethanol in PBS at –20°C for 2 hours) and additional washes, cells were incubated with propidium iodine (PI) staining solution (20 µg/ml PI, 0.1% Triton X-100 and 0.2 mg/ml RNase A in PBS) for 30 minutes at 25°C. The parameters of forward scatter (FSC>400) and CD41^+^ signals (FL1>10^2^) were used to gate and measure the percentage of polyploid megakaryocytes (among total 2×10^5^ total bone marrow cells). DNA content was analyzed using a flow cytometer and CellQuest program (Becton-Dickinson).

### Statistics

Means, standard deviations, and statistics for quantifiable data were calculated using Microsoft Office Excel 2003 for Windows. Groups were compared using two-tailed Student’s *t* test, and *p-*values less than 0.05 were considered significant.

## Supporting Information

Figure S1 **Treatments of protective antigen (PA) did not induce apoptosis of human cord blood-derived megakaryocytes.** A 16-day course of megakaryocytic differentiation for human cord blood-derived CD34+ mononuclear cells was performed. LT was treated at day 12 and then analyzed at day 16. Parameters of the cell size (FSC) and cell granularity (SSC) are indicated (A). By flow cytometry analysis, anti-CD61 antibodies were use to investigate the megakaryocytic marker CD61 expression (B); and AnnexinV-APC (C) and active caspase-3 antibodies (D) were used to investigate the apoptotic changes of LT-treated cells. Summarized events were shown on (E). Data are reported as mean ± standard deviation (SD) and represent at least 3 independent experiments.
(TIF)

Figure S2 **PA treatments did not induce thrombocytopenia in mice.** (A) The experimental outline used. The platelet counts of mice at 22, 44, and 66 hours after PA (1.25 mg/kg) and saline injections are shown (B). Saline challenged mice were used as negative controls.
(TIF)

Figure S3 **Post-treatments of TPO prolonged the survival time of LT-challenged mice.** (A) The experimental outline. The survival rate of mice injected with TPO, LT, or LT plus TPO appears in (B). The asterisk (*) marks in (A) and (B) indicate the starting time point for recording survival rate. C57BL/6J mice were retro-orbitally injected with recombinant murine TPO (0.25 µg/mouse, in 250 µl saline) twice every 24 hours after injection of a lethal dose of LT (1.5 mg/kg in 250 µl saline, retro-orbital injection). Experimental groups using either saline challenge or TPO alone without further LT challenge served as controls. The survival times of mice were recorded after the LT injection.
(TIF)

Methods S1 **Supplemental experimental procedures.**
(DOC)
