# Supplementary figures and images for: Suppressive Effects of Anthrax Lethal Toxin on Megakaryopoiesis
Source: PLoS One. 2013 Mar 21;8(3):e59512. doi: 10.1371/journal.pone.0059512 (PMC3605335; doi:10.1371/journal.pone.0059512)

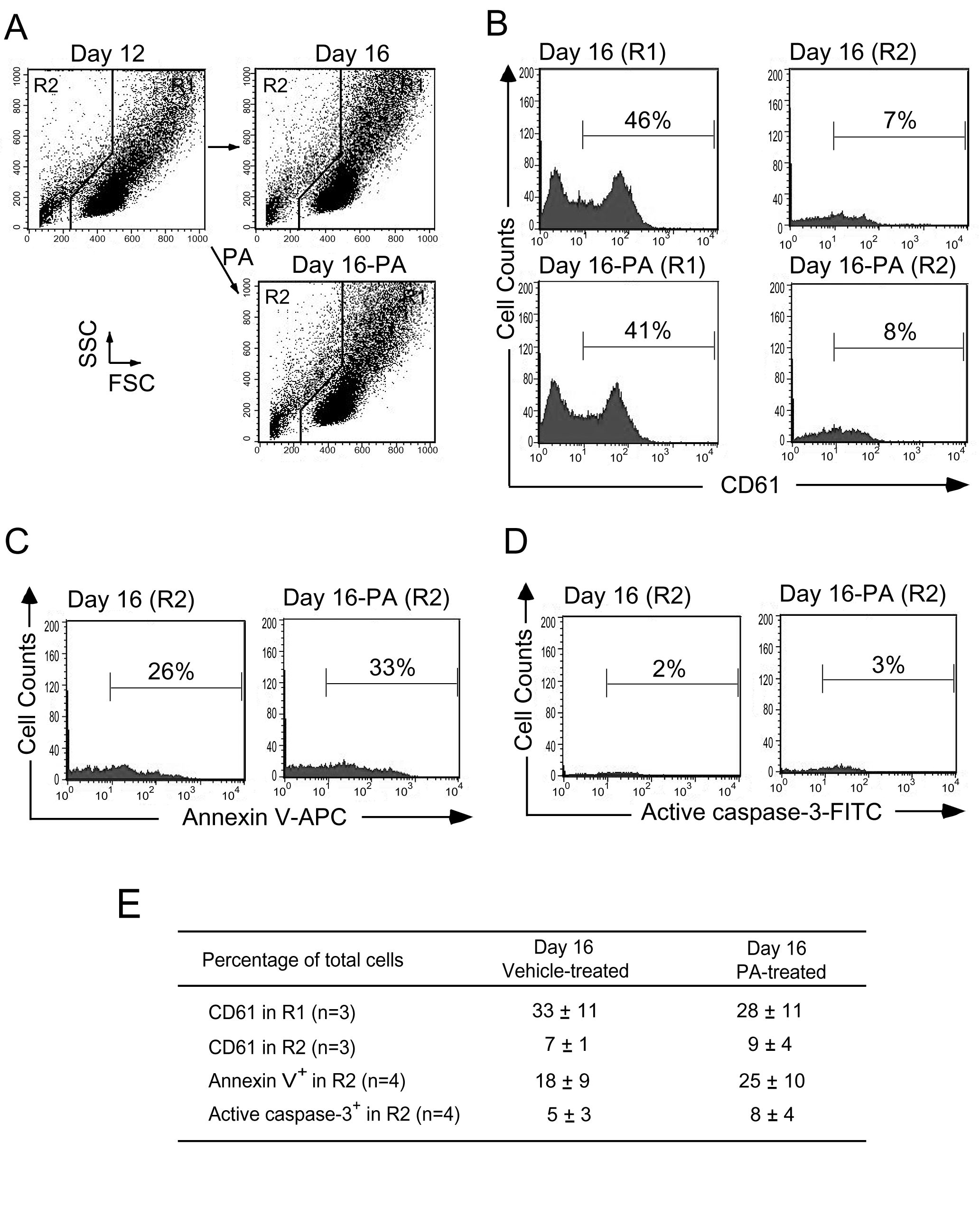

Supplement: Figure S1 — Treatments of protective antigen (PA) did not induce apoptosis of human cord blood-derived megakaryocytes. A 16-day course of megakaryocytic differentiation for human cord blood-derived CD34+ mononuclear cells was performed. LT was treated at day 12 and then analyzed at day 16. Parameters of the cell size (FSC) and cell granularity (SSC) are indicated (A). By flow cytometry analysis, anti-CD61 antibodies were use to investigate the megakaryocytic marker CD61 expression (B); and AnnexinV-APC (C) and active caspase-3 antibodies (D) were used to investigate the apoptotic changes of LT-treated cells. Summarized events were shown on (E). Data are reported as mean ± standard deviation (SD) and represent at least 3 independent experiments. (TIF) [file pone.0059512.s001.tif]

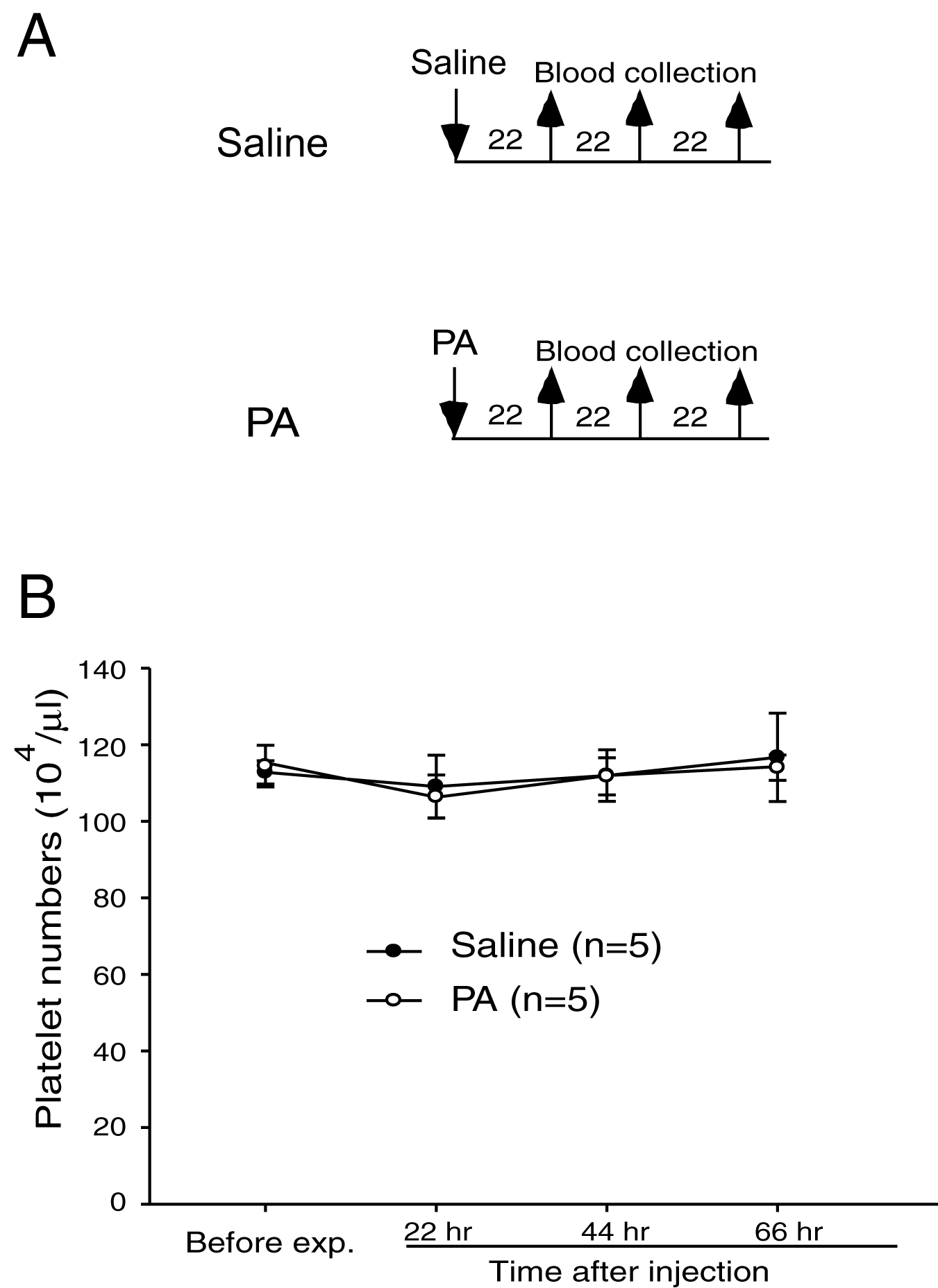

Supplement: Figure S2 — PA treatments did not induce thrombocytopenia in mice. (A) The experimental outline used. The platelet counts of mice at 22, 44, and 66 hours after PA (1.25 mg/kg) and saline injections are shown (B). Saline challenged mice were used as negative controls. (TIF) [file pone.0059512.s002.tif]

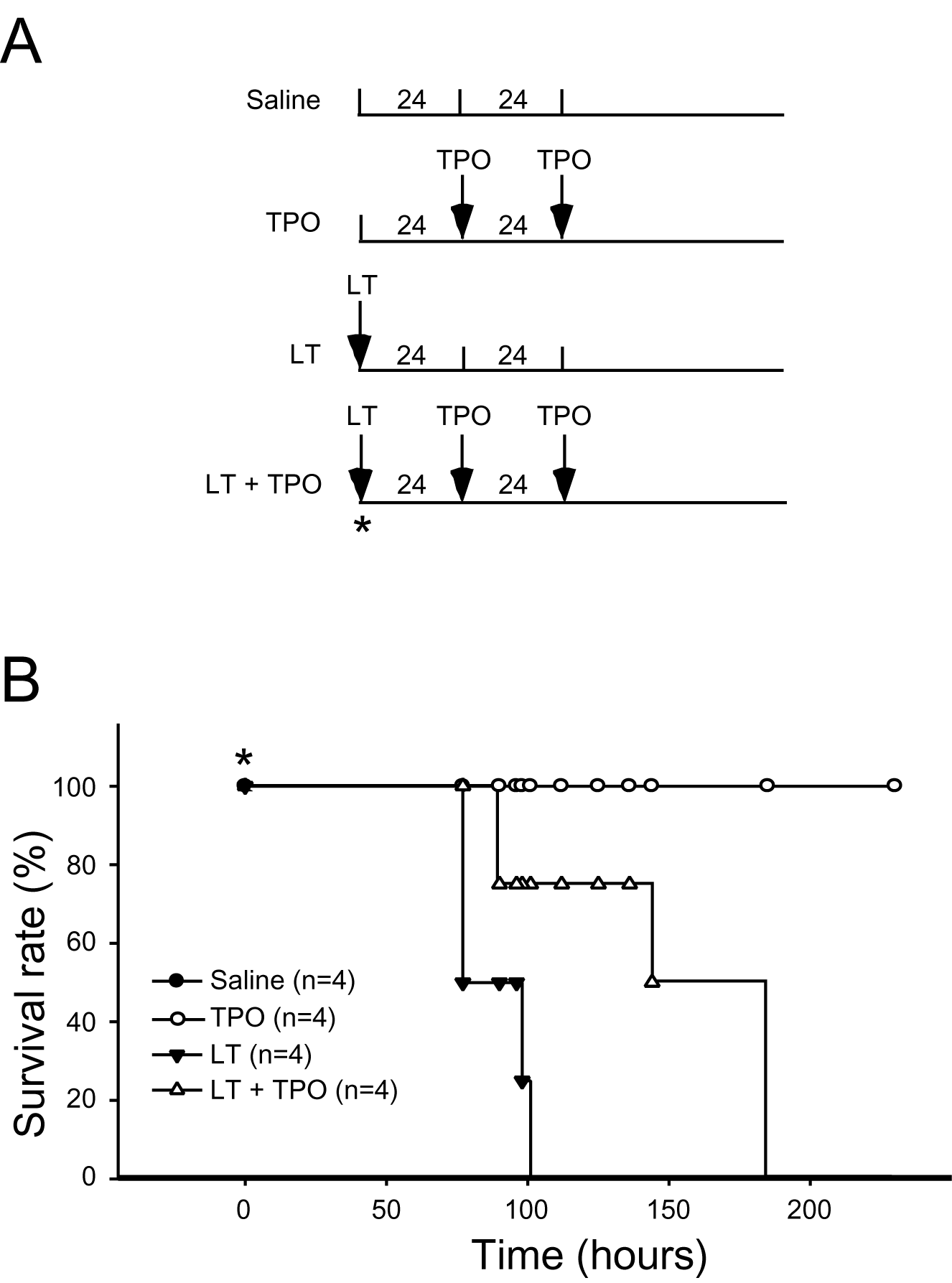

Supplement: Figure S3 — Post-treatments of TPO prolonged the survival time of LT-challenged mice. (A) The experimental outline. The survival rate of mice injected with TPO, LT, or LT plus TPO appears in (B). The asterisk (*) marks in (A) and (B) indicate the starting time point for recording survival rate. C57BL/6J mice were retro-orbitally injected with recombinant murine TPO (0.25 µg/mouse, in 250 µl saline) twice every 24 hours after injection of a lethal dose of LT (1.5 mg/kg in 250 µl saline, retro-orbital injection). Experimental groups using either saline challenge or TPO alone without further LT challenge served as controls. The survival times of mice were recorded after the LT injection. (TIF) [file pone.0059512.s003.tif]
