# Supplementary material for: Suppressive Effects of Anthrax Lethal Toxin on Megakaryopoiesis
Source: PLoS One. 2013 Mar 21;8(3):e59512. doi: 10.1371/journal.pone.0059512 (PMC3605335; doi:10.1371/journal.pone.0059512)
Supplement: Methods S1 — Supplemental experimental procedures. (DOC) [file pone.0059512.s004.doc]

**Supplemental experimental procedures**

**Megakaryocyte in vitro culture and flow cytometry assay**

Following the manufacturer’s instructions, human CD34^+^ mononuclear cells were isolated from cord blood using a CD34 MicroBead kit (Miltenyi Biotec, Bergisch Bladbach, Germany). To expand the cell number, CD34^+^ cells were cocultured with mesenchymal stem cells (MSC) from Wharton’s jelly of umbilical cords. At this stage, cells were cultured in IMDM (Gibco) containing 10% fetal bovine serum (FBS) (Biological Industries, Kibbutz Beit Haemek, Israel), 10 ng/ml recombinant human (rh) TPO (PeproTech), 20 ng/ml rh IL-3 (PeproTech), 30.5 ng/ml rh stem cell factor (SCF) (PeproTech), and 22.3 ng/ml rh Flt-3 ligand (FL) (PeproTech) for 4-5 days . To induce megakaryocytic differentiation, CD34^+^ cells (5 × 10^5^ in one 6-well dish) were cultured at 37°C in 5% CO2 and 100% humidity without MSC, using differentiation medium-IMDM supplemented with 3% FBS, 2 mM l-glutamine, 100 U/l penicillin, 100 mg/ml streptomycin, and cytokines including 50 ng/ml rh TPO, 7.5 ng/ml rh IL-6 (PeproTech), 1 ng/ml rh SCF, and 13.5 ng/ml interleukin 9 (IL-9) (PeproTech) . To sustain the differentiation for 16 days, differentiation medium was freshly prepared and renewed every 4 days. PA (10 ng/ml) was added to the medium at day 12. After 4-day PA treatments, cells were analyzed by flow cytometry. The levels of megakaryocytic specific surface markers were determined by fluorescein-isothiocyanate (FITC)-conjugated anti-human CD61 (eBioScience, San Diego, CA) antibodies. To analyze apoptotic cells, allophycocyanin (APC)-conjugated Annexin V (BD Pharmingen, CA, USA) and FITC-conjugated anti-active form caspase-3 (BD Pharmingen) antibodies were used. Digitized results were then analyzed and quantified using the CellQuest program (Becton-Dickinson).

**Platelet counts measurement after PA challenge**

C57BL/6J mice (males, 8-10 weeks old) were retro-orbitally injected with *B. anthracis*-derived PA (1.25 mg/kg in 250 μl saline, retro-orbital injection). Groups injected with normal saline (diluent) were served as controls. To measure the platelet counts of mice, 50 μl of blood samples were collected retro-orbitally 22, 44, and 66 hours after injection, and subsequently mixed with 450 μl anticoagulant citrate dextrose (ACD)-containing diluents (1:9). The hematopoietic parameters were determined using an automated hematology analyzer (KX-21, Sysmex Corporation, Kobe, Japan).

**Post-treatments of TPO prolong surviving time of LT-treated mice**

C57BL/6J mice (males, 8-10 weeks old) were purchased from the National Laboratory Animal Center (Taipei, Taiwan) and maintained in a specific pathogen-free condition in the experimental animal center of Tzu Chi University. The research methods involving the experimental mice were approved by the Institutional Animal Care and Use Committee at Tzu Chi University (Approval ID: 97060; Project: Molecular characterization of megakaryocytic differentiation). C57BL/6J mice were retro-orbitally injected with recombinant murine TPO (CytoLab/PeproTech Asia, Rehovot, Israel) (0.25 μg/mouse, in 250 μl saline) twice at 24 and 48 hours after injection of a lethal dose of LT (1.5 mg/kg in 250 μl saline, retro-orbital injection). Experimental groups injected with either saline or TPO alone without further LT challenge were served as controls. The mortality and survival time of mice were recorded after the LT challenge.

**References**

1. Yao C-L, Liu C-H, Chu IM, Hsieh T-B, Hwang S-M (2003) Factorial designs combined with the steepest ascent method to optimize serum-free media for ex vivo expansion of human hematopoietic progenitor cells. Enzyme and Microbial Technology 33: 343-352.

2. Bakhshi T, Zabriskie RC, Bodie S, Kidd S, Ramin S, et al. (2008) Mesenchymal stem cells from the Wharton's jelly of umbilical cord segments provide stromal support for the maintenance of cord blood hematopoietic stem cells during long-term ex vivo culture. Transfusion 48: 2638-2644.

3. Cortin V, Pineault N, Garnier A (2009) Ex vivo megakaryocyte expansion and platelet production from human cord blood stem cells. Methods Mol Biol 482: 109-126.
